# A systematic review, and meta-analysis, examining the prevalence of price promotions on foods and whether they are more likely to be found on less-healthy foods

**DOI:** 10.1017/S1368980019004129

**Published:** 2020-06

**Authors:** Asha Kaur, Thomas Lewis, Veronika Lipkova, Santhushya Fernando, Mike Rayner, Richard A Harrington, Wilma Waterlander, Peter Scarborough

**Affiliations:** 1 Centre on Population Approaches for Non-Communicable Disease Prevention, Nuffield Department of Population Health, University of Oxford, Old Road Campus, Oxford OX3 7LF, UK; 2 Washington Singer Laboratories, Perry Road, University of Exeter, Exeter EX4 4QG, UK; 3 Medical Sciences Divisional Office, University of Oxford, Level 3, John Radcliffe Hospital, Oxford OX3 9DU, UK; 4 Ministry of Health, Nutrition and Indigenous Medicine, Ministry of Health, Colombo 10, Colombo 01000, Sri Lanka; 5 NIHR Oxford Biomedical Research Centre, Oxford University Hospitals NHS Foundation Trust, Oxford, UK; 6 Amsterdam UMC, University of Amsterdam, Department of Public Health, Amsterdam Public Health Research Institute, Meibergdreef 9, Amsterdam, The Netherlands

**Keywords:** Price promotions, Marketing, Food, Nutrition, Diet

## Abstract

**Objective:**

There are concerns that price promotions encourage unhealthy dietary choices. This review aims to answer the following research questions (RQ1) what is the prevalence of price promotions on foods in high-income settings, and (RQ2) are price promotions more likely to be found on unhealthy foods?

**Design:**

Systematic review of articles published in English, in peer-review journals, after 1 January 2000.

**Setting:**

Included studies measured the prevalence of price promotions (i.e. percentage of foods carrying a price promotion out of the total number of foods available to purchase) in retail settings, in upper-mid to high-income countries.

**Participants:**

‘Price promotion’ was defined as a consumer-facing temporary price reduction or discount available to all customers. The control group/comparator was the equivalent products without promotions. The primary outcome for this review was the prevalence of price promotions, and the secondary outcome was the difference between the proportions of price promotions on healthy and unhealthy foods.

**Results:**

Nine studies (239 344 observations) were included for the meta-analysis for RQ1, the prevalence of price promotions ranged from 6 % (95 % CI 2 %, 15 %) for energy-dense nutrient-poor foods to 15 % (95 % CI 9 %, 25 %) for cereals, grains, breads and other starchy carbohydrates. However, the I-squared statistic was 99 % suggesting a very high level of heterogeneity. Four studies were included for the analysis of RQ2, of which two supported the hypothesis that price promotions were more likely to be found on unhealthy foods.

**Conclusions:**

The prevalence of price promotions is very context specific, and any proposed regulations should be supported by studies conducted within the proposed setting(s).

A poor diet is a leading risk factor for ill health with unhealthy diets associated with more than 12 million deaths globally^(1)^. Within the EU more than a million deaths are associated with dietary risk factors such as diets high in sodium, low in wholegrain and low in fruits and vegetables^(2)^.

The price of foods and drinks (henceforth referred to as ‘foods’) is an important factor for consumers when making dietary choices^(3)^. Price promotions are a type of marketing that involves displaying a temporary reduction in price (e.g. 10 % of the price) and/or ‘volume-based’ promotions where an increase in volume is offered at a reduced price (e.g. buy-one-get-one-free offers). In the 2018 Childhood Obesity strategy, the UK Government announced that it intends to ban volume-based price promotions on unhealthy foods (foods high in fat, sugar and salt)^(4)^. Price reductions will be exempt from the legislation as volume-based price promotions require the consumer to buy more to receive the discount and ‘…have been shown to specifically encourage and stimulate over-purchasing to a larger extent’ compared with price reductions^(page 9,(5))^.

In a study commissioned by Public Health England, the impact of price promotions *on purchases* was estimated through the analysis of purchase data from 30 000 households in the UK between 2013 and 2015. One finding was that price promoted foods were associated with a growth in sales of 22 %, even after considering consumer stockpiling and the subsequent delayed repurchasing^(6)^. The same study also found that 40 % of household expenditure was on price promoted food and drink and that Britain had the highest prevalence of price promotions (i.e. the proportion of foods carrying price promotions) in Europe. However, the current study was based on purchase data collected by a consumer panel, and therefore the estimated prevalence of price promotions is based on the percentage of *sales*, rather than on the percentage of foods that are *available* for consumers to purchase. This distinction is important, since the shopping environment in which consumers make purchasing decisions is better characterised by food availability data than food sales data. Furthermore, there is some evidence that a greater availability of goods is associated with greater consumption, and similarly reducing availability is associated with reduction in consumption in other arenas (e.g. alcohol and tobacco regulation and consumption^(7,8)^.

Sales-based scanner data are collected through scanning the barcode of products purchased by participating households or individuals (‘panel members’). A strength of scanner-based data is that it can produce thorough datasets containing details for a large number of products, often from multiple retailers, and often over long periods of time^(9)^. A study of price promotions using scanner data was conducted by Nakamura *et al.* ^(10,11)^ using data collected by Kantar. The scanner data included purchase data from 26 986 households in the UK in 2010. The analysis in the current study was restricted to products that had been purchased by one household in each week of the study period (i.e. 52 weeks, *n* 11 323 products). Nakamura *et al.* found that healthier and less-healthy foods had a similar prevalence of price promotions amongst purchased foods. However, controlling for the reference price, price discount rate and brand-specific effects, the sales uplift arising from price promotions was larger in less healthy than in healthier categories. Nakamura *et al*. also found that the unhealthy foods were more likely to have larger proportional discounts (sometimes referred to as a deeper price promotion).

There are concerns that price promotions are encouraging unhealthy diets and that they may also be contributing to social inequalities in health and/or diets^(12–14)^. Price promotions could worsen diets by encouraging additional consumption and/or by shifting purchases from healthier foods to less-healthy foods (or food categories)^(15)^. Conversely, price promotions could also improve diets if they are encouraging purchases of healthier foods.

It can be difficult to identify how many and what types of foods carry price promotions as there are multiple channels from which to purchase foods, for example, through retailers (e.g. supermarkets, convenience stores etc.) or through food service settings (e.g. canteens, restaurants, markets etc.). The food supply can be assessed through analyses of sales-based data (e.g. scanner data) and through surveys of foods available to purchase in retail settings.

A limitation of some scanner-based studies is that in order for a product to be captured in the data, it must be purchased (and scanned) by a panel participant – foods that do not carry price promotions that have not been purchased by a participant are not captured. The purchase-based nature of the sample may introduce a bias leading to an overestimation of the prevalence of price promotions if foods with price promotions are more likely to be purchased than foods that do not carry them.

Another method to assess the food supply is to conduct surveys or audits of retail settings. For example, Potvin Kent *et al.* ^(16)^ collected data on all of the ready-to-eat breakfast cereals available to purchase in the five largest supermarkets in Canada and found that 77 % of price promoted breakfast cereals were categorised as ‘Less healthy’ by the UK Nutrient Profile Model^(17)^. An advantage of such surveys is that they measure foods available to purchase. However, this method of collecting data can be time and resource intensive. As a result, such studies are often restricted to either a small number of foods or food categories.

Previous reviews concerning price promotions have examined the impact of price promotions on purchases and/or consumption^(18,19)^ and how consumer responses to price changes may differ according to personal characteristics^(20)^. However, we are not aware of any systematic reviews that have examined the prevalence of price promotions using data on food availability.

## Objectives

The primary research question for this systematic review is ‘what is the prevalence of price promotions on foods?’ The secondary research question is ‘are price promotions more likely to be found on healthier or less-healthy foods?’ The setting of this systematic review is upper-mid to high-income countries.

## Methods

The protocol for this review is available in Appendix A. The protocol was developed by the authors to answer the following research questions ‘what is the prevalence of price promotions on foods in high-income settings’ and ‘are price promotions more likely to be found on unhealthy foods?’. The finalised protocol contained the author-agreed definitions for the study populations (‘foods’), interventions (price promotions), control/comparator (foods from the same population that are not on price promotion) and outcomes (prevalence of price promotions). The protocol detailed the methods and search strategy to be used in this review. The definitions and eligibility criteria were referred to during the development of the search strategy and for the eligibility assessment of papers.

### Identification of studies

We searched the following databases: PubMed, Scopus and Web of Science. We also examined all of the articles identified as ‘similar articles’ whilst searching on PubMed. The bibliography/reference section of included studies was then manually searched for relevant articles. The search strategy included terms relating to or describing interventions (e.g. price promotions, dietary intervention), potential study outcome measures (e.g. nutrition, diet, obesity) and settings (food retail, supermarkets, discrete choice). We also used Medical Subject Heading (MeSH) searches based on terms relating to or describing obesity. The search strategy was developed by examining relevant papers known to the authors including, Powell *et al.* ^(21)^, Taillie *et al.* ^(22)^, Nakamura *et al.* ^(23)^, Ayala *et al.* ^(24)^, Caspi *et al.* ^(25)^, Ravensbergen *et al.* ^(26)^ and Thornton *et al.* ^(27)^. The initial searches were conducted in May 2018, with an updated search conducted in June 2019. All search terms are provided in Appendix B.

The returned hits from the searches were imported into Endnote v7. One researcher removed all of the duplicates and titles that were not related to price promotions or food. The remaining titles and abstracts were screened by two researchers with any disagreements in inclusion proceeding to the full text review. Articles were included at the full paper screen if they met the inclusion criteria or if more information was required before making a decision. A validity assessment was conducted on 10 % of the titles at the title screen stage to check for agreements/disagreements between the two researchers. Any disagreements were discussed and arbitrated by a third researcher.

The data extraction sheet was compiled in Excel (the column headings are listed in Appendix C). The data extracted from included studies contained: study details e.g. (year of publication, study design); sample details (e.g. methods, setting); study results, type of price promotion (proportional discount, multi-purchases etc.) and analyses (e.g. statistical analyses performed, whether adjusted for confounding factors etc.). Full-text review and data extraction were conducted by a team of four researchers such that every paper in the full-text review was reviewed by two researchers with arbitration in the case of disagreement by a different researcher.

### Selection of studies

To be eligible for inclusion studies must have been published in English in a peer-reviewed journal after 1 January 2000.

An article was eligible for inclusion if it reported data on the prevalence of consumer-facing price promotions in a retail setting in an upper mid- to high-income country. These countries were identified though the World Bank’s categorisation of country incomes^(28)^. Country-level income was used as a criterion as supermarkets are the main point of purchase in these countries and less so in lower to lower-mid income countries^(29)^.

An article was excluded if:

- estimates for the prevalence on price promotions were based purely on sales data,
- measurement consisted only of promoted products and therefore does not provide enough data to calculate prevalence of promotions (e.g. as in flyers and circulars),
- it measured the impact of price promotions on sales (or purchasing intent) but did not report the prevalence of price promotions,
- it manipulated the prevalence of price promotions in an artificial setting (e.g. virtual supermarkets, choice experiments) without presenting real-world data on the prevalence of price promotions,
- the price promotion was presented in a food service setting (e.g. restaurants, canteens etc.) and not a retail setting,
- the price reduction was only available to certain sub-groups of the population or based on the shopper’s characteristics or previous shopping behaviour (e.g. student discounts, store loyalty points),
- the price promotion was retailer facing (i.e. trade promotions), not consumer facing.

### Definitions

Population: The populations for this review were the sets of foods available for purchase in specific setting(s), from which a (sometimes comprehensive) sample of foods had been examined in the study. ‘Foods’ was defined as foods and non-alcoholic beverages intended for human consumption. Non-food items (e.g. household supplies), alcoholic beverages (and low alcohol and/or alcohol-free equivalents) and non-food items that are intended for human consumption (e.g. tobacco, vaping substances etc.) were not considered in this review.

Duplication: For studies that measured the prevalence of price promotions at multiple time points, we calculated an average for the time points. If multiple studies used the same dataset (i.e. identical time point(s), location(s) and food categories), the most informative study was included, and the other(s) were marked as duplicates and not included for analyses. Where studies used different subsets of the same dataset (e.g. identical time point(s), location(s), but different food categories), both studies were included for data extraction.

Intervention: ‘Price promotion’ was defined as a consumer-facing temporary price reduction or discount available to all customers. This definition means that multi-buy offers (e.g. buy-one-get-one free, three for the price of two etc.) are included, but discounts based on personal characteristics or previous shopping behaviour (e.g. student discounts, store loyalty points) are not.

Outcome: The primary outcome was the prevalence of price promotions (i.e. the percentage of foods that carry a price promotion out of the total number of foods examined). The secondary outcome was the difference in the prevalence of price promotions in ‘healthier’ and ‘less healthy’ food categories. For this, we did not apply a definition of ‘healthier’ or ‘less healthy’, but reported results on the basis of definitions found in the included studies.

The unit of analysis for this review was the number of eligible foods included in the study for which price promotion data were available. The control group/comparator was the equivalent products without promotions.




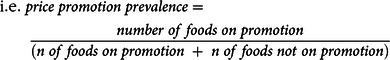




Data synthesis, analysis, risk of bias assessment

For the primary research question (what is the prevalence of price promotions?), we conducted a meta-analysis on the included studies. We used a random effects model for the current analysis as we expected variation in types of price promotion, year of studies, type of retail setting and the sampling and data collection methods. Data were presented by food category and all categories combined. The food groupings were based upon those used in the UK Eatwell Guide^(30)^. Foods were grouped into one of the following categories:

- Beverages (e.g. soft drinks, fruit juices, bottled water),
- Cereals, grains, breads and other starchy carbohydrates,
- Dairy foods,
- Energy dense, nutrient poor foods (e.g. confectionery, crisps/potato chips)
- Meat, fish and eggs.

To explore explanations for heterogeneity in the results, we conducted multi-level meta-regressions (outcomes nested in studies). The outcome for these regression analyses was the log-transformed (to ensure normality) proportion of foods on promotion in each food category and in each study. The exposure variables, each included in univariate models, were geography (North America *v*. elsewhere), study type (studies that included data from more than one time point *v*. others) and food group (beverages, cereals, dairy, meat, fruit/vegetables and energy dense nutrient poor).

For the second research question (Are price promotions more likely to be found on healthier or less-healthy food categories?), we expected multiple definitions of ‘healthier’/ ‘less healthy’ and multiple statistical methods to be employed. Therefore, we did not attempt a meta-analysis on these data. Instead, a two-step approach was used. In the first step, a sign-test indicated whether the prevalence was higher for the less-healthy foods than for the healthier foods. In the second step, we reported whether or not the observed differences were statistically significant using a threshold of *P* < 0·05.

For the risk of bias assessment, we used Durant’s^(31)^ individual quality criteria, adapted for our research questions (Table 1). This tool was identified from a systematic review which examined criteria that are used to assess the quality of cross-sectional and longitudinal studies^(32)^.

**Table 1:** Adapted quality of included studies criteria

Criteria	Assessment
Definition of population	Are the criteria for inclusion of shops described?
	Are the criteria for inclusion of foods described?
Sampling strategy	Was the sample drawn randomly from the population or is the sample a complete audit of the population?
Description of sample	Has the study sample been clearly described in terms of: date(s) of data collection, sample size of foods; geographic region; sample size of shops?
Definition of outcome variable (price promotion)	Has the study provided a definition of ‘price promotions’?
	Has the method of categorising foods with ‘price promotions’ been validated (including inter-rater reliability)?
Definition of potential exposure variables	Has the study provided a definition of any included exposure variables (e.g. food categories, healthiness of foods)?
	Has the method of categorising foods for exposure variables been validated (including inter-rater reliability)?

Planned analyses by type of price promotion were not possible due to a lack of data in the literature.

## Results

### Search results

**Fig. 1:**
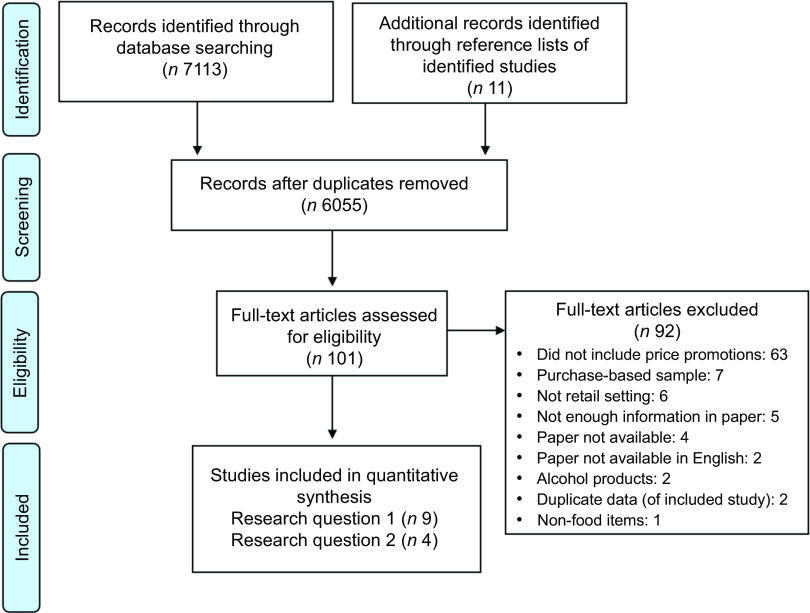
Prisma flow chart

The PRISMA diagram for this review is presented in Fig. 1 ^(33)^. In total, 7113 articles were identified through the database searches, once the duplicates were removed (1058 articles) 6055 abstracts remained. In total, sixty-eight papers were included at the full paper screening, fifty-seven of these titles were identified through the database searches and eleven references were identified by manually searching the reference sections of the included studies.

The most common reason for exclusion at the full paper screen was the lack of price promotion data that met the inclusion criteria (*n* 63), the use of price promotions in a non-retail setting (*n* 6), for example, studies that examined price promotions used in flyers/circulars (e.g.^(34)^), or examined price promotions offered to retailers (trade promotions) and not promotions offered to consumers (i.e.^(35)^). There were five studies that were excluded because there was not enough information presented in the paper to calculate the prevalence of price promotions^(36–40)^. There were seven studies that contained purchase-based data relating to price promotions that could not be transformed/collapsed to calculate the availability of products^(22,23,41–45)^.

There were nine studies included for the meta-analysis for research question 1: Arce-Urriza *et al.* ^(46)^, Black *et al.* ^(47)^, Bronnmann and Asche^(48)^, Empen *et al.* ^(49)^, Glauben *et al.* ^(50)^, Lucan *et al.* ^(51)^, Potvin Kent *et al.* ^(16)^ and Powell *et al.* ^(21)^. Three of these papers were included for research question 2: Black *et al.* ^(47)^, Potvin Kent *et al.* ^(16)^, Powell *et al.* ^(21)^ and Zorbas *et al.* ^(52)^.

A summary of the included studies is presented in Table 2. Five of the included studies were conducted in Europe: three in Germany^(48–50)^, one in Spain^(46)^ and one in the UK^(47)^. The four remaining studies were conducted in the USA^(21,51)^, Canada^(16)^ and Australia^(52)^.

**Table 2:** Characteristics of included studies

First author (year)	Setting(s)	Study design*	Setting	Food groups	Number of stores	Number of Foods	Number of time-points†	Number of observations‡	Eligible for RQ2?§
Arce-Urriza *et al.* ^(46)^	Loyalty card data (3416 customers) from a leading national retailer in Spain. Capturing purchases of 1 litre orange juice made in-store and/or offline between May–November 2007. Promotional data presented as the number of days a product was price-promoted over study period.	Longitudinal	Supermarkets (online and in-stores)	Fruit juices	>500 stores + and/or online	7	180 d	1260	No
Black (2014)^(47)^	Store audits conducted between July 2010–June 2011, assessing the availability of and promotions on twelve food products in 601 food retailers in Hampshire, UK. Store types audited included large stores, premium stores, discount stores, and ‘world’ stores.	Cross-sectional	Supermarkets, grocery stores and corner/convenience stores	Meat, fish and eggs; dairy products; energy dense-nutrient poor; cereals, grains, bread and other starchy carbohydrates; beverages; fruit and vegetables	601	12	1	5168	Yes
Bronnmann and Asche ^(48)^	Scanner data containing household purchases of frozen fish products, between 2000–2010, across multiple retailers in Germany	Longitudinal	Supermarkets	Meat, fish and eggs	Not reported	568	132 months	74 976	No
Empen *et al.* ^(49)^	Retail scanner data of purchases of ready-to-eat breakfast cereals, between January 2000–December 2001, from 108 German retail stores.	Longitudinal	Supermarkets	Cereals, grains, bread and other starchy carbohydrates	108	23	104 weeks	1 729	No
Glauben *et al.* ^(50)^	Retail scanner data containing purchases of perishable dairy products (twelve leading brands for four types of dairy products), over 2 years, across multiple retailers in Northern Germany.	Longitudinal	Supermarkets, discounters, consumer markets	Dairy products	17	48	104 weeks	32 817	No
Lucan *et al.* ^(51)^	Store audits conducted between June and September 2011, assessing all fresh fruit and vegetable products sold in 44 stores (supermarkets and other store types) in the Bronx, NY, USA.	Cross-sectional	Grocery stores	Fresh fruit and vegetables	44	NA‖	1	3 363	No
Potvin Kent *et al.* ^(16)^	Store audits conducted over 4 weeks assessing shelf-facings, promotional displays, and costs of breakfast cereals in five of the largest supermarkets in Ottawa/Gatineau, Canada.	Cross-sectional	Supermarkets	Cereals, grains, bread and other starchy carbohydrates	5	225	1	588	Yes
Powell *et al.* ^(21)^	Store audits conducted in 2010, 2011 and 2012, assessing availability of and promotions on 44 foods sold in 8959 food stores, across 468 communities in the USA.	Longitudinal	Supermarkets, grocery stores and limited service stores	Meat, fish and eggs; energy dense nutrient poor; cereals, grains, bread and other starchy carbohydrates; beverages; fruit and vegetables	8 959	44	3 years	165 342	Yes
Zorbas *et al.* ^(52)^	Audits of non-alcoholic beverages available to purchase through two online supermarkets, conducted every week between November 2016–November 2017, in Australia	Longitudinal	Supermarkets (online)	Sugar sweetened beverages, artificially sweetened beverages, milk, flavoured milk drinks, fruit juice, water	2	15	50 weeks	1942	Yes

* ‘Longitudinal’ refers to data collected on the same products and/or from the same store for more than one time point.

† ‘Time-points’ refers to the number of occasions for which data which were collected, for example, weekly sales data.

‡ ‘Observations’ refers to the number of eligible food observations on which the prevalence estimate was based, (e.g. for longitudinal studies this is the number of foods surveyed at each of the time points combined).

§ ‘RQ2’ refers to the secondary research question: ‘are price promotions more likely to be found on healthier or less-healthy foods?’.

‖ ‘NA’ refers to studies where data was collected on all foods available to purchase, rather than studying a set of defined/indicator foods.

### Types of studies

Four studies were one-off audits of stores, two of which were conducted in the US (Lucan *et al.* ^(51)^, Powell *et al.* ^(21)^), one in Canada (Potvin Kent *et al.* ^(16)^) and one in the UK (Black *et al.* ^(47)^). Another study consisted of weekly audits (*n* 50) of two online supermarket retailers in Australia (Zorbas *et al.* ^(52)^). The three German studies examined scanner data^(48–50)^ and the Spanish study used loyalty card data^(46)^ (note that data for the these studies were provided on the total number of unique foods at each time point, allowing us to calculate prevalence of price promotions based on availability rather than sales). The period of time captured with the scanner and loyalty card data varied between 6 months (Arce-Urriza *et al.* ^(46)^) and 11 years (Bronnmann and Asche^(48)^).

The four studies that contained store audits varied in the types of foods studied and the setting. One study, Powell *et al.* ^(21)^ examined a set of indicator foods (*n* 44) across 8959 stores across 468 communities in the USA. Black *et al.* ^(47)^ had a similar approach and examined a set of twelve indicators foods across 601 stores in Hampshire, UK. Whereas, the remaining three studies examined all of the products within a single food category: Potvin Kent *et al.* ^(16)^ examined all of the ready-to eat breakfast cereals (*n* 225) available to purchase in five of the largest supermarket chains in Canada, Lucan *et al.* ^(51)^ examined all of the fresh fruit and vegetables available to purchase in forty-four stores in the Bronx, NY, USA, and Zorbas *et al*.^(52)^ examined non-alcoholic beverages available to purchase from the websites of two major Australian supermarket chains.

### Types of settings and foods

Supermarkets were the most common setting of the included studies, with all but one study examining price promotions in this setting^(51)^. Two studies studied online supermarkets^(46,50)^. Four studies^(21,46,47,50)^ involved multiple settings: Arce-Uriza *et al.* ^(46)^ looked at products available to purchase in both brick-and-mortar supermarkets and through online supermarkets; Black *et al.* ^(47)^ studied supermarkets, grocery stores and convenience stores; Glauben *et al*.^(50)^ studied supermarkets, discounter stores and consumer markets and Powell *et al.* ^(21)^ studied supermarkets, grocery stores and limited service stores.

The methods used to identify the stores varied: Potvin Kent *et al.* ^(16)^ chose the largest retailers in the area, Lucan *et al.* ^(51)^ identified the forty-four stores by systematically surveying the area within half a mile of all of the farmers’ markets in the area. The farmers’ markets were identified by examining government business lists for the area. Powell *et al*.^(21)^ also used lists and business directories to identify stores in 468 communities. These communities were selected as the public middle-school and high-school students resident in each community were nationally representative samples. Powell *et al.*’s^(21)^ audit involved collecting data in 2010, 2011 and 2012, in each year, the stores were identified through combining multiple lists of businesses. The stores were eligible for auditing if they sold foods and this was assessed through phone calls to each store. In addition to this, stores were identified through fieldwork by data collectors. Black *et al.* ^(47)^ identified 606 stores through Council Food Safety Registers and online business directories, and fieldworkers then collected data from 601 stores between July 2010 and June 2011. Zorbas *et al.* ^(52)^ studied the websites of the two leading supermarkets chains in Australia. Between November 2016 and November 2017, data were collected on non-alcoholic beverages available to purchase through the websites. Beverages that required preparation before consumption (e.g. tea, coffee etc.) were excluded, although an exception was made for cordial drinks on the basis that it is commonly consumed by children.

The scanner data studies (Bronnmann and Asche^(48)^, Empen *et al.* ^(49)^ and Glauben *et al.* ^(50)^) were all conducted in Germany but studied different products. Bronnmann and Asche^(48)^ examined all frozen fish products (*n* 528) purchased by the panel, across multiple retailers, over an 11-year period. Using MaDaKom data from 2001 to 2002, Empen *et al.* ^(49)^ examined all ready-to-eat breakfast cereal purchases from 108 retailers (1729), and Glauben *et al.* ^(50)^ looked at the twelve leading brands for four types of dairy products (milk, *n* 4167, yoghurt *n* 12 447, cheese *n* 9736, butter *n* 6467) over 2 years. Arce-Urriza *et al.* ^(46)^ examined 6 months of customer loyalty card data of a single national retailer containing data on orange juice purchases made by consumers who purchased food both online and in-store.

### Identification of price promotions

There were differences in how studies identified price promotions and how much detail was reported in the paper. Of the scanner data studies, Bronnmann and Asche^(48)^ identified foods as being on promotion or not, but it is unclear how this was defined. In the Arce-Urriza *et al.* ^(46)^ study, products were categorised as being on price promotion when the retail price per product was less than the usual retail price. Empen *et al.* ^(49)^ defined price promotions as a reduction of at least 5 % of the usual price, whereas Glauben *et al*.^(50)^ reported that price promotions were defined as a temporary (less than 4 weeks) price reduction of 5 % or more (relative to the modal price per year).

The store audits recorded the presence of price promotions on the day(s) of data collection although there were still differences in how this was characterised. Lucan *et al*. ^(51)^ recorded both the usual sale price of foods and (for foods on price promotion) the discounted price of foods. Black *et al*.^(47)^ took a similar approach but also reported that price promotions made on the day (e.g. due to food spoilage and/or quality) were excluded. Potvin Kent *et al*.^(16)^ also recorded both the sale price and usual retail price, but excluded price reductions that were present in each week of data collection (i.e. if a food had the same price in weeks 1, 2, 3 and 4, it was not considered as being price promoted). Powell *et al.* ^(21)^ identified price promotions through notices on shelf tags that indicated a temporary price reduction (e.g. ‘sale’, ‘special’, ‘save’, ‘price cut’, ‘deal’ etc.) and/or had a different colour to usual shelf tags. Shelf tags that referred to permanent price reductions (e.g. ‘everyday low price’) were excluded^(21)^. Similarly, Zorbas *et al.* ^(52)^ defined price promotions as temporary price reductions and excluded ‘everyday low price’ promotions. Zorbas *et al.* ^(52)^ collected data on a weekly basis for each non-alcoholic beverage that was on sale at a price lower than the regular selling price. For the first 26 weeks of the study, data were collected manually and recorded in Excel. For the second half of the study, data were recorded from the retailer websites using automated web-scraping methods. Mid-way through the study (May 2017), a complete audit of all non-alcoholic beverages (i.e. both price-promoted and non-promoted beverages) was conducted manually. These data were combined with the weekly audits to calculate the proportion of products on price promotion. Promotions were categorised as either a ‘price promotion’ (referring to a temporary price reduction) and/or a ‘multi-buy’ promotion which was defined as ‘a price promotion that required consumers to purchase more than one unit to receive the discount…’^(page 2, 50)^.

### Prevalence of price promotions

The meta-analysis, based on 239 344 observations from nine studies, showed a very high amount of heterogeneity in the prevalence of price promotions. The I-squared statistic was 99 % suggesting a very high level of heterogeneity – this statistic was also over 95 % for each of the food groups by which the meta-analysis was stratified. This suggests that price promotion strategies vary to a great deal between food groups, retailers, settings and geographical locations.

**Fig. 2:**
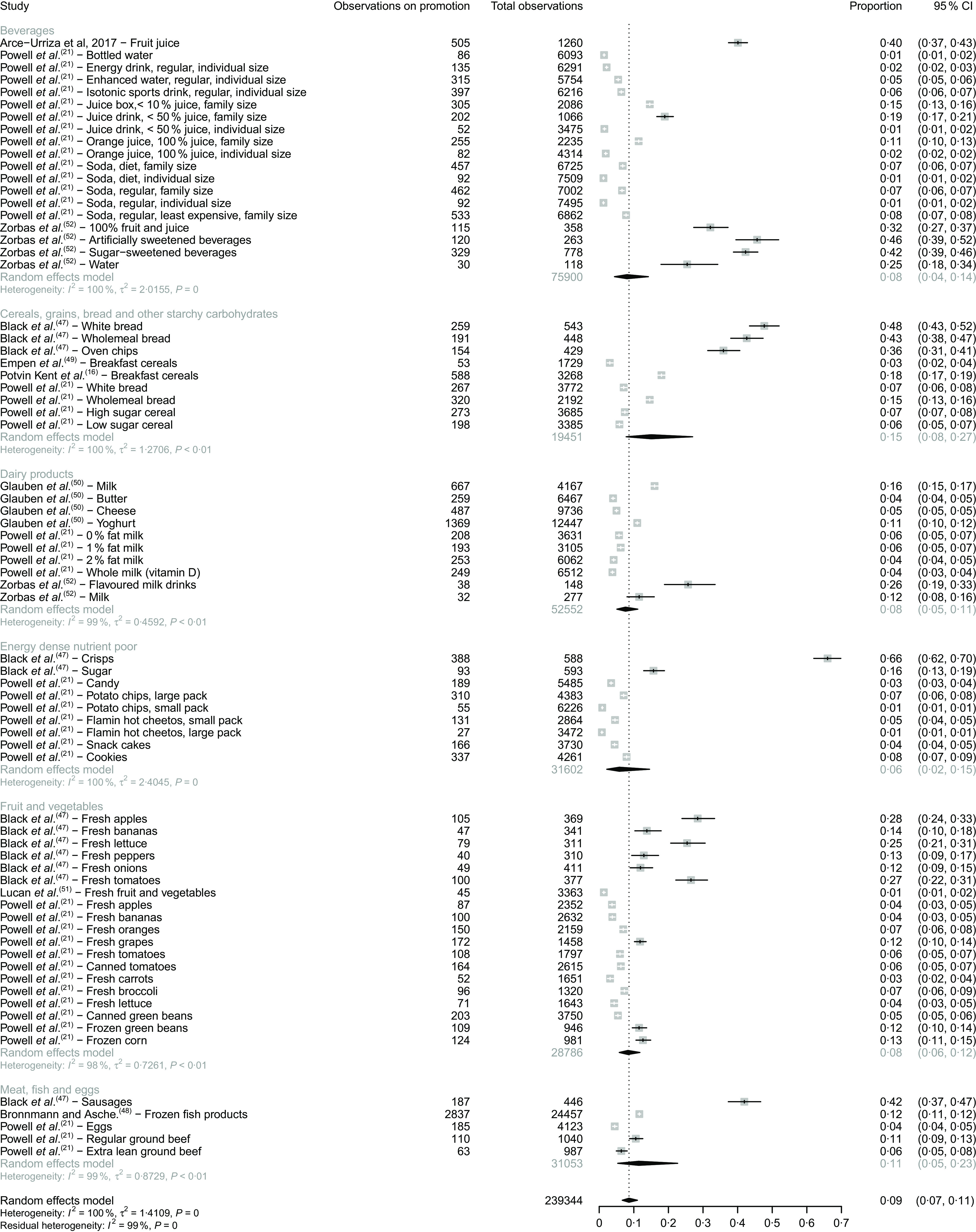
Forest plot of the prevalence of price promotions by food category

The prevalence of price promotions ranged from 6 % (95 % CI 2 %, 15 %) for the energy-dense nutrient-poor group to 15 % (95 % CI 9 %, 25 %) for the cereals, grains, breads and other starchy carbohydrates group (Fig. 2). The prevalence of price promotions was similar for the fruit and vegetables group (8 %, 95 % CI 6 %, 11 %), the dairy products group (8 %, 95 % CI 5 %, 11 %) and the beverages group (8 %, 95 % CI 4 %, 14 %).

There was some variation within each food group, for example, with the fruit and vegetables group the prevalence of price promotions ranged from 1 % (95 % CI 1 %, 2 %) for fresh fruit and vegetables (reported by Lucan *et al.* ^(51)^) to 28 % (95 % CI for 24 %, 33 %) for fresh apples (reported by Black *et al.* ^(47)^). A similar pattern was observed within the cereals, grains, breads and other starchy carbohydrates group, which ranged from 3 % of breakfast cereals (95 % CI 2 %, 4 %, reported by Empen *et al.* ^(49)^) to 48 % of white bread (95 % CI 43 %, 52 %) reported by Black *et al.* ^(47)^.

Three studies (Black *et al.* ^(47)^, Arce-Urriza *et al.* ^(46)^ and Zorbas *et al.* ^(52)^) found a much higher prevalence of price promotions than the other six included studies. Arce-Urriza *et al.* ^(46)^ used average time on promotion as an outcome measure and found that orange juice products were, on average, on promotion 40 % of the time (505 d/1260 d, 95 % CI 37 %, 43 %). We conducted an additional meta-analysis omitting the current study, and the prevalence estimate was similar when the study was included (7 %, 95 % CI 6 %, 9 %, I-squared 99 %). Black *et al.* ^(47)^ conducted an audit of food stores in the UK and examined the use of price promotions on twelve food products and found that overall 32 % of observed foods (95 % CI 31 %, 34 %, *n* 1692/5166) had a price promotion (data via personal communication). Zorbas *et al.* ^(52)^ conducted weekly audits of non-alcoholic beverages available to purchase through two online retailers in Australia to measure the prevalence of price reduction promotions and volume-based promotions for each retailer. We consolidated the results from each retailer to compare the prevalence of price reductions and the prevalence of volume-based promotions and found that 28 % (95 % CI 26 %, 30 %) of non-alcoholic beverages had a price reduction promotion and 6 % (95 % CI 5 %, 7 %) of non-alcoholic beverages had a volume-based promotion. Price reductions were more prevalent in artificial and sugar-sweetened beverages (36 %, 95 % CI 30 % 42 % and 33 %, 95 % CI 30 %, 37 %, respectively) than for flavoured milks (24 %, 95 % CI 17 %, 31 %), fruit juices (27 %, 95 % CI 22 %, 31 %), water (22 %, 95 % CI 15 %, 30 %) and (non-flavoured) milk (11 %, 95 % CI 7 %, 15 %). For the meta-analysis, we combined the results by retailer and by promotion sub-type and found that overall, 34 % (95 % CI 32 %, 36 %) of non-alcoholic beverages had a price promotion. Artificially sweetened beverages had a higher prevalence of price promotions (46 %, 95 % CI 40 %, 52 %) than sugar-sweetened beverages (42 %, 95 % CI 39 %, 46 %), flavoured milk drinks (26 %, 95 % CI 19 %, 33 %) and water (25 % 95 % CI 18 %, 33 %). Milk beverages had the lowest lower prevalence of price promotions (12 %, 95 % CI 8 %, 15 %).

### Stores and brands

Arce-Urriza *et al.* ^(46)^ reported large differences in the average number of days that different brands of orange juice were on promotion. The prevalence ranged from 12 % to 61 % depending on the brand studied with store branded products on price promotion 41 % of the time. Empen *et al.* ^(49)^ also found brand differences. Empen *et al.* ^(49)^ reported that the overall average prevalence of price promotions was 3 %; when stratified by brand the average prevalence ranged from 0·5 % (Koln) to 6·7 % (Nestle). The remaining two brands Kellogg’s and Dr Oetker had an average prevalence of price promotions of 2·6 % and 4·5 %, respectively. Zorbas *et al.* ^(52)^ studied two retailers and found similar patterns in the distribution of price reduction promotions by retailers (but did not report results for specific brands). Powell *et al.* ^(21)^ found that supermarkets had the highest prevalence of price promotions (13 % of foods sampled), but that foods most likely to be price promoted varied by store type. However, across all store types, fresh fruits and vegetables had a lower prevalence of price promotions than other food categories. The remaining studies^(16,47,48,50,51)^ did not report the prevalence of price promotions stratified by brand or store type.

Stratifying by geographic region found that the Australian study reported a higher prevalence of price promotions (29 %, 95 % CI 20 %, 40 %, from 1942 observations, 1 study) than the European studies^(46–50)^ (20 %, 95 % CI 14 %, 28 %, from 65 429 observations, five studies) and the studies conducted in North America^(16,21,51)^ (5 %, 95 % CI 4 %, 6 %, from 171 973 observations, three studies). A multi-level meta-regression found no statistical difference between these regions (*P* < 0·08). However, there is collinearity between the geographic region and study type: the Australian and North American studies were audits of stores, whereas three of the European studies involved scanner data^(46,48–50)^ and one of the studies was an audit of a store^(47)^. The meta-analysis by data collection method found that scanner studies reported a slightly higher prevalence (10 %, 95 % CI 5 %, 18 %, from 60 263 observations, four studies) than the studies that were audits (9 %, 95 % CI 6 %, 11 %, from 179 081 observation, five studies). However, this difference was not statistically significant (*P* < 0·90).

The meta-analysis by study design found that the cross-sectional studies reported a higher prevalence (23 %, 95 % CI 15 %, 35 %, from 11 797 observations, three studies) than longitudinal studies (7 %, 95 % CI 5 %, 8 %, from 227 547 observations, six studies). However, the multi-level meta-regression found that this difference was not statistically significant (*P* < 0·95).

Whilst nine studies were included in the meta-analysis, one study contributed 69 % of the observations used in the analyses (Powell *et al.* ^(21)^ – 165 342 observations). When we omitted the Powell *et al.* ^(21)^ study, the prevalence rate increased to 20 % (95 % CI 14 %, 27 %).

We were unable to conduct a meta-analysis examining the difference in prevalence between volume-based promotions and price-reductions promotions due to a lack of data in the literature as a lone study^(52)^ measured the prevalence in different types of price promotions.

The risk of bias assessment of the current study (Table 3) found that generally the included studies were of a good quality. Where appropriate, all studies reported the inclusion criteria for stores, foods and geographic region. None of the studies used random sampling as they all either examined a complete sample of foods or all foods within a selected food category.

**Table 3:** Risk of bias assessment of included studies

Study	Description of store inclusion criteria	Description of foods inclusion criteria	Foods sampling type	Description of size of food sample (weight/volume)	Description of geographic region	Description of store sample size	Definition of price promotions	Validation of categorisation of promotions	Definition of exposure variable(s)	Validation of categorisation of exposure variable(s)
Arce-Urriza *et al.* ^(46)^	No	Met	Complete	Met	Met	Na	Met	Unmet	Met	Na
Black *et al*.^(47)^	Met	Met	Complete	Unmet	Met	Met	Unmet	Unmet	Met	Na
Bronnmann and Asche^(48)^	Unmet	Met	Complete	Met	Met	Met	Unmet	Na	Met	Na
Empen *et al.* ^(49)^	Met	Met	Complete	Unmet	Met	Met	Met	Met	Met	Na
Glauben *et al.* ^(50)^	Met	Met	Complete	Unmet	Net	Met	Met	Met	Met	Na
Lucan *et al.* ^(51)^	Met	Met	Complete	Na	Met	Met	Met	Met	Met	Met
Potvin Kent *et al.* ^(16)^	Met	Met	Complete	Unmet	Met	Met	Met	Unmet	Met	Met
Powell *et al.* ^(21)^	Met	Met	Complete	Met	Met	Met	Met	Unmet	Met	Unmet
Zorbas *et al.* ^(52)^	No	Met	Complete	Unmet	Met	Na	Met	Met	Met	Met

### Are price promotions on healthier or less-healthy foods?

We found mixed evidence that price promotions were more prevalent on less-healthy foods (Table 4). Powell *et al.* ^(21)^ used two comparisons to assess ‘healthiness’: comparing the price promotion prevalence between food categories (e.g. snacks and sweets to fruits and vegetables) and by comparing foods by their nutritional content, that is, fat (e.g. lean meat *v.* regular meat, fat-free milk *v.* 1–2 % fat milk) and sugar (cereals higher sugar and cereals lower sugar).

**Table 4:** Sign test of difference between prevalence of promotions in healthy and unhealthy foods

Study	Methods for comparison	Food groups	Prevalence of price promotions (%)	95 % CIs	Direction/significance of difference*
Black *et al.* ^(47)^	Comparison of 7 healthier foods and 5 less-healthy food items	Healthier foods:			
		Peppers	13	9, 17	
		Tomatoes	27	22, 31	
		Lettuce	25	21, 30	
		Onions	12	9, 15	
		Apples	28	24, 33	
		Bananas	14	10, 17	
		Wholemeal bread	43	38, 47	
		Less-healthy foods:			
		Oven chips	36	31, 40	
		Sausages	42	37, 47	
		Crisps	66	62, 70	
		Sugar	16	13, 19	
		White bread	48	43, 52	
		Overall:			
		Healthier foods	24	22, 25	–
		Less-healthy foods	42	40, 43	
Potvin Kent *et al*.^(16)^	Comparison of breakfast cereals using the UK Nutrient Profile Model to define foods as healthier and less healthy	Healthier breakfast cereals	27	23, 31	**++**
		Less-healthy breakfast cereals	18	17, 19	
Powell *et al*.^(21)^	Comparison of healthier and less-healthy food categories	0–2 % fat milk	5	5, 5	**++**
		Whole fat milk	4	3, 4	
		100 % juices	5	5, 5	–
		<50 % juices	8	8, 9	
		Bottled water (plain)	1	1, 2	–
		Bottled water (sweetened)	5	5, 6	
		Diet sodas	4	4, 4	–
		Non-diet sodas†	5	5, 5	
		Fruits and vegetables	6	6, 6	**++**
		Snacks foods‡	4	4, 4	
		Lean beef	6	5, 8	–
		Regular beef	11	9, 12	
		Low sugar cereal	6	5, 7	–
		High sugar cereal	7	7, 8	
		Wholemeal bread	15	13, 16	**++**
		White bread:	7	6, 8	
		Overall:			
		Healthier	5·2	5·1, 5·4	No difference
		Less healthy	5·1	5·0, 5·2	
Zorbas *et al*.^(52)^	Comparison between policy-relevant food categories and nutritional contribution of category to diet	*All price promotions*			
		Healthier beverages:			
		Water	25	18, 33	
		Milk	12	8, 15	
		Flavoured milk	26	19, 33	
		100 % fruit/vegetable juices	32	27, 37	
		Less-healthy beverages:			
		Sugar-sweetened beverages	42	39, 46	
		Artificially sweetened beverages	46	40, 52	
		Overall:			
		Healthier beverages	24	21, 27	–
		Less-healthy beverages	43	40, 46	
		*Price reduction promotions*			
		Healthier beverages:			
		Water	22	15, 30	
		Milk	11	7, 15	
		Flavoured milk	24	17, 31	
		100 % fruit/vegetable juices	27	22, 31	
		Less-healthy beverages:			
		Sugar-sweetened beverages	33	30, 37	
		Artificially sweetened beverages	36	30, 42	
		Overall:	28	26, 30	
		Healthier beverages	21	18, 24	–
		Less-healthy beverages	34	31, 37	
		*Volume-based promotions*			
		Healthier beverages:			
		Water	3	−14, 21	
		Milk	0	−11, 12	
		Flavoured milk	1	−15, 17	
		100 % fruit/vegetable juices	5	−5, 15	
		Less-healthy beverages:			
		Sugar-sweetened beverages	9	2, 16	
		Artificially sweetened beverages	10	−2, 21	
		Overall:	6	2, 10	
		Healthier beverages	3	−4, 9	–
		Less-healthy beverages	9	3, 15	

* ‘--’ Less-healthy foods more likely to be on promotion (*P* < 0·05); ‘-’ Less-healthy foods more likely to be on promotion (*P* > 0·05); ‘+’ More healthy foods more likely to be on promotion (*P* > 0·05); ‘++’ More healthy foods more likely to be on promotion (*P* < 0·05).

† Food group includes energy and sports drinks.

‡ Food group includes potato chips, cookies, snack cakes.

In our analyses of the Powell *et al.* ^(21)^ study (using data provided in the paper), we merged the results for the different store types and found that overall there was no statistically significant difference in the prevalence of price promotions between the healthier and less-healthy foods. In our analyses of the Black *et al.* ^(47)^ data (provided by the author), we calculated the total number of healthy and less-healthy foods observed in the study and the number that were on price promotion (within each of those categories) and found that price promotions were more prevalent for less-healthy foods (42 %, 95 % CI 40 %, 43 %) than for healthier foods (24 %, 95 % CI 22 %, 25 %).

Potvin Kent *et al.* ^(16)^ used the UK Nutrient profile model to categorise breakfast cereals as healthier and less healthy. When examining which category had the highest proportion of price promotions, healthier or less-healthy breakfast cereals, we found that 27 % (95 % CI 23 %, 31 %) of healthier breakfast cereals had a price promotion compared with 18 % (17 %, 19 %) of less-healthy breakfast cereals. However, due to the different quantity of healthier and less-healthy breakfast cereals available, the authors point out that the majority of price promotions were actually found on less-healthy cereals.

Zorbas *et al.* ^(52)^ categorised beverages into four policy-relevant categories: sugar-sweetened beverages, artificially-sweetened beverages, flavoured milk and 100 % fruit juice and milk and water. Zorbas *et al.* ^(52)^ characterised the flavoured milk and 100 % fruit and vegetable juices as typically having a higher nutritional value than other sugar-sweetened beverages. Similarly, Zorbas *et al.* ^(52)^ describe the milk and water categories as having ‘nutritional importance’ to the diet. Using the data presented in the paper, we merged the results for these healthier and less-healthy food categories and found support for the hypothesis that price promotions were more likely to be observed on less-healthy foods; 43 % (95 % CI 40 %, 46 %) of the less-healthy beverages carried price promotions compared with 24 % (95 % CI 21 %, 27 %) of the healthier beverages. Price reduction promotions were more prevalent (28 %, 95 % CI 26 %, 30 %) than volume-based promotions (6 %, 95 % CI 5 %, 7 %). The prevalence of price reductions was higher for less-healthy beverages (34 %, 95 % CI 31 %, 37 %) than for healthier beverages (21 %, 95 % CI 18 %, 24 %).

## Discussion

This systematic review uncovered a very high level of heterogeneity among the included studies due to the different food groups, settings and methods of the included studies. For this reason, the pooled prevalence estimated derived from the meta-analysis is of limited use. This review uncovered a relatively low prevalence of price promotions: with the exception of three studies (Arce-Urriza *et al.* ^(46)^, Black *et al.* ^(47)^ and Zorbas *et al*.^(52)^), all of the results that we identified estimated price promotion prevalence rates of 20 % or lower. We found no consensus in the very limited literature with regard to whether price promotions are more likely to be found on healthy or unhealthy food and drink. Results from the only study conducted in the UK^(47)^ showed considerable differences in the prevalence of price promotions between unhealthy (42 %, 95 % CI 40 %, 43 %) and healthy foods (24 %, 95 % CI 22 %, 25 %). For another study conducted in the USA^(21)^, there were directly competing results for different comparisons – for example, price promotions were more likely to be found on wholemeal bread than white bread (15 % *v.* 7 %), but also more likely to be found on regular beef than on lean beef (11 % *v.* 6 %).

In 2018, the UK Government announced that it intends to ban the use of volume-based price promotions (e.g. where the consumer has to buy more to receive the discount such as buy-one-get-one-free) on unhealthy foods^(4)^. Unfortunately, in our analyses, we were unable to differentiate between volume-based promotions and price-reductions promotions due to a lack of data in the literature. A lone study examined the difference in types of price promotion, Zorbas *et al.* ^(52)^ measured the prevalence of price promotions on non-alcoholic beverages available to purchase from to online retailers in Australia. In our analyses of data presented in the current study, we found that price promotions were more likely to be found on less-healthy beverages (43 %, 95 % CI 40 %, 46 %) than healthier beverages (24 %, 95 % CI 21 %, 27 %) and that less-healthy beverages had a higher prevalence of price reduction promotions (34 %, 31 %, 37 %) than healthier beverages (21 %, 95 % CI 18 %, 24 %). Whilst the current study has some limitations (the denominator on which the prevalence estimates were made was based on one full audit conducted mid-way through the study), this was a comprehensive and high-quality study of a large food category (non-alcoholic beverages) available to purchase through two supermarket websites on a weekly basis over a year. It is also the only study that our review identified that allows for analyses by sub-type of price promotion.

Overall, the findings from this review offer support for UK government’s policy as a key finding of this review is that the prevalence of price promotions is very context-specific, with European studies reporting a higher prevalence of price promotions than North American studies, and the only UK-based study identified in this review found a much higher prevalence of price promotions on unhealthy foods than for healthier foods^(47)^. However, the Australian study^(52)^ found that volume-based offers were much less prevalent than price reduction offers. Similar research is needed urgently in the UK to measure the potential impact of restricting volume-based promotions on unhealthy foods.

A poor diet is a leading cause of ill-health^(1)^; therefore, if price promotions have any impact on purchasing behaviour, then it could be argued that they should be restricted on unhealthy foods even if price promotions are not disproportionately found on unhealthy products or if the overall prevalence of price promotion is low. Regulation, or at a minimum more monitoring, would be justified particularly when considering the high prevalence of price promotions reported in studies using purchase-based data (i.e. examining the proportion of *purchased* products on price promotion, as opposed to the proportion of *available* products on price promotion (e.g. ^(23,53)^). For example, analysis presented in the UK’s Sugar Reduction policy documents found that 41 % of UK household food expenditure was on price promoted food and drink, whereas the equivalent expenditure in other European countries (e.g. Germany, France and Spain) was approximately 20 %^(6,53)^. The over-representation of price promotions in this purchase-based dataset (relative to the estimate from this review) suggests that UK consumers may be more sensitive to price promotions and/or that price promotions are more prevalent in the UK.

Price promotions on unhealthy foods may be particularly unfavourable if they have a long-shelf life as stockpiling price-promoted perishable items such as fresh fruit and vegetables may not necessarily lead to greater consumption (e.g. due to food waste arising from spoilage)^(18)^.

Further research is needed to measure seasonal trends. The included studies often identified price promotions through observed changes in the items selling price. However, the prices of foods fluctuate, for example, the prices of fruits and vegetables fluctuate due to seasonal effects (e.g.^(54,55)^). More research is also needed to identify whether there are seasonal variations in the prevalence of price promotions. Future research should also investigate regional variations in price promotions. Within the UK, the Competition Commission reported that there may be regional variations in retailers’ use of price promotions^(56,57)^. However, we were unable to investigate this further as none of the included studies examined regional differences in the prevalence of price promotions.

This is the first systematic review, of which we are aware, that has examined studies of the prevalence of price promotions on food and drinks available to purchase in retail settings. A recent (non-systematic) review^(58)^ also highlighted the lack of research on the prevalence of price promotions, but cite recent research that found that price promotions are highly prevalent amongst samples of purchased foods.

In our systematic review, the data extraction stages were comprehensive, and each study was double-extracted to ensure consistencies in data extraction. However, due to the time constraints, we were unable to search for relevant data in grey literature and/or policy documents. We searched GreyLit.org and did not find any relevant documents that met our inclusion criteria; however, this database ceased to be updated in 2017. We are aware of at least three documents from the UK and Ireland, but each would have been excluded at the full paper screening stage. The data presented in the UK Government’s Sugar Reduction Strategy^(53)^ and that presented by NHS Scotland^(59,60)^ would not be eligible for inclusion as they both use purchase-based data. An audit conducted in Ireland would not be eligible as the audit only examined items that were price promoted so could not be used to calculate the prevalence of price promotions as foods without price promotions were not audited^(61)^. We are aware of unpublished data that would be relevant to this review: Waterson, Dobson and Seaton^(62)^ collected data from four UK supermarkets (Tesco, Sainsbury’s, Ocado, Asda) between August 2010 and August 2011. The nutritional composition of foods carrying price promotions was assessed using the UK’s front of pack traffic light labelling criteria^(63)^ to define foods as having high, medium and low levels of fat, saturated fat, sugar and salt. The study found that, on average, relative to foods that did not carry a price promotion, foods that carry price promotions were more likely to have high levels of sugar, but not more likely to have ‘high’ levels of fat, saturated fat and salt. The study also found that foods carrying price reduction offers were skewed towards unhealthy items, whereas volume-based promotions were more likely to be found on healthier items. However, more information would be required to incorporate these results into our study. For example, it is unclear whether alcoholic beverages have been excluded, and we were unable to analyse this further due to time constraints.

This review did not include studies of low-income countries. This is a limitation as supermarkets in low-income countries have increased rapidly^(29)^, and there is some evidence (e.g. from a high-income country^(21)^) that supermarkets have a higher prevalence of price promotions than other, smaller retail settings.

Another limitation of this review is that we did not have a consistent definition of ‘healthy’ and ’unhealthy’, instead we used the definitions used in the included studies. One study (Black *et al.*)^(47)^ compared healthy foods (e.g. apples and bananas) and less-healthy foods (e.g. crisps and sausages), whilst other studies^(21,52)^ compared healthier food categories (e.g. fruits and vegetables) and less-healthy food categories (e.g. snack foods). However, such comparisons may not show the variation of nutritional quality within food categories (e.g. dried fruit v. fresh fruit). A single study^(16)^ used an objective measure of healthiness (The Ofcom Nutrient Profile model^(17)^).

This review found differences in the methods and definitions used in the included studies. There is a need for standardised methods and definitions such as those proposed by the International Network for Food and Obesity/non-communicable Diseases Research, Monitoring and Action Support (INFORMAS)^(64)^.

This review highlights the need for a health impact assessment of the UK Government’s proposed policy to ban volume-based price promotions on unhealthy foods. However, before this can be achieved, more research is required to assess the use of price promotions across multiple food categories and retail settings along with estimates to quantify the impact of price promotions on purchases. Any regulations should be supported by and evaluated with studies conducted within the proposed setting(s) to assess whether regulation would be an effective response to public health concerns. Future research should incorporate this data in modelling studies that estimate the impact of removing price promotions on health outcomes.

## Supplementary material

For supplementary material accompanying this paper visit https://doi.org/10.1017/S1368980019004129. click here to view supplementary material
