# Supplementary material for: A systematic review, and meta-analysis, examining the prevalence of price promotions on foods and whether they are more likely to be found on less-healthy foods
Source: Public Health Nutr. 2020 Mar 25;23(8):1281–96. doi: 10.1017/S1368980019004129 (PMC7196736; doi:10.1017/S1368980019004129)
Supplement: Supplementary file 1 [file S1368980019004129sup.zip › S1368980019004129sup001.pdf]

Appendix A: Protocol for 'a systematic review, and meta-analysis, examining the prevalence of price promotions on foods and whether they are more or less likely to be found on unhealthy foods.

CONTEXT

Poor diet is leading cause of poor health with lower socioeconomic groups more likely to have a poor diet and experience diet-related ill-health. Price promotions are temporary reductions in price or additional products at a reduced rate or for free. Previous research suggests that lower socioeconomic status groups are more sensitive to price promotions and that consumers whom most favour price promotions are more likely to be overweight. There have been concerns that price promotions on unhealthy food is contributing to poor diet and in 2018 the UK government announced that it intends to ban price promotions.

Price promotions may promote unhealthy dietary choices if they encourage excessive, additional consumption and/or shift purchasing habits from healthier foods to less healthy foods. But, previous estimates of the prevalence of price promotions have often been based upon purchase-based datasets (such as consumer panel datasets) where a price promotion is only recorded if a product has been purchased by a participating household whilst it is on price promotion [(1)]. These data sources may be bias towards foods that are on price promotion (if products that are on price promotion are more likely to be purchased). Therefore, it remains unclear what proportion of foods are available to purchase carry price promotion and whether healthier or less healthy food s are more likely to have them.

REVIEW QUESTIONS

What is the prevalence of price promotions on foods available to purchase in food retail settings, in upper mid to high-income countries?

Are price promotions more likely to be found on healthy or less healthy foods?

SEARCHES

We will search the following databases: PubMed, Scopus and Web of Science. We shall also examine the articles that are identified as 'similar articles' whilst searching on PubMed. We will search the bibliography and reference sections of articles included at the full paper screen.

The search strategy will include words relating to or describing interventions (e.g. price promotions, dietary intervention), potential study outcome measures (e.g. nutrition, diet, obesity), settings (food retail, supermarkets, discrete choice). We will also use Medical Subject Heading (MeSH) searches based on terms relating to or describing obesity.

The search strategy was developed by examining relevant papers known to the authors. These papers were; Powell et al (2016, [(2)]), Taillie et al (2017, [(3)]), Nakamura et al (2015, [(1)]), Ayala et al, (2017, [(4)]), Caspi et al (2017, [(5)]), Ravensbergen et al (2015, [(6)]), and Thornton et al (2017, [(7)]).

To be eligible for inclusion articles must be published in English, in peer-review journals, after the 1st of January 2000.

TYPES OF STUDY TO BE INCLUDED

An article will be included if it:

- examines the prevalence of price promotions of foods and non-alcoholic beverages available to purchase in a food retail setting in an upper-mid to high-income country. We will use the World Bank categorisation of country incomes [(8)]. We will only look at upper-mid to high-income countries as supermarkets have been identified as the main point of purchase for household food consumption in these countries (Reardon and Hopkins, 2007 [(9)]).
- is an observational study, or cross-sectional study, or audit, which contains data from a single time point or collects longitudinal data relating to the prevalence of price promotions
- presents consumer panel or scanner data that can be aggregated so that the unit of analyses is the foods available to purchase (rather than foods purchased by consumers). Often articles present scanner or consumer panel data where the unit of analysis is the foods purchased by consumers, whilst this is a purchase-based sample (and therefore may already be biased towards foods with price promotions) the article shall be considered for inclusion if data can be collapsed so that the unit of analyses is foods available to purchase. This is under the assumption that such data sets are designed to be representative of the population and that every product available to purchase will be purchased at least once by at least one participant, so aggregating data at the food level will be an accurate representation of all foods available to purchase.

An article will be excluded if it:

- does not collect or present data relating to the prevalence of price promotions in a food retail setting in an upper-mid to high income country - reviews, editorials etc. will not be included unless they present new data.
- measures the prevalence of price promotions on non-food or drink products such as household products,
- only measures the prevalence of price promotions on alcoholic beverages, or alcohol-free versions of alcoholic beverages, or non-food items that are intended for human consumption e.g. tobacco, vaping etc.
- measures the prevalence of price promotions in food service settings (e.g. restaurants, fast-food outlets) rather than in a food retail setting (e.g. supermarkets, food stores, online food shopping),
- examines the impact of price promotions on sales or consumer preferences or intents, without reporting the (real-world) prevalence of price promotions
- examines the impact of price promotions (or their prevalence) in an artificial setting such as a choice experiment or virtual supermarket,
- presents consumer panel or scanner data where the unit of analyses is foods purchased and data cannot (or is not available to) be aggregated so that the unit of analyses is the foods available to purchase.

CONDITION OR DOMAIN BEING STUDIED

Price promotions on food and non-alcoholic beverages available to purchase in a food retail setting, in upper mid to high-income countries.

PARTICIPANTS/POPULATION

The population is the sample of foods examined in the study. For this review 'food' is defined as food and non-alcoholic beverages (or alcohol-free versions of alcoholic beverages) intended for human consumption. The definition does not include alcoholic beverages or non-food items that are intended for human consumption e.g. tobacco, vaping. If data is presented for non-food items or alcoholic beverages then we will only include the study if this data can be isolated from the food and non-alcoholic beverages.

We are interested in foods available to purchase in food retail settings (e.g. supermarkets, stores, etc.) and not a food service setting (e.g. restaurants, canteens, service stations). This is because biological and/or physiological factors (e.g. hunger, appetite, cravings) or pragmatic factors (e.g. constraints on time available to consume or prepare food) may play a larger role in choices made in food service settings particularly of foods intended to be consumed at the place of purchase. Food service settings may be more opportunistic than food retail settings, for example offering price reductions at different times of the day to reduce food wastage or spoilage. Food service settings tend to offer different types of price promotions that may not be applicable to food in the retail sector e.g. super-sized pricing, all-you-can eat buffets, free-refills.

If an article presents data on the prevalence of price promotions at multiple time points then we will consider them as separate estimates unless the time points are within the same 24-hour period (for example, in studies which measure product availability in-store and/or measures the prevalence of out-of-stock products, empty shelves etc.).

INTERVENTION(S)/EXPOSURE(S)

We will define price promotions as temporary reductions in price. 'Price' refers to the amount consumers pay for the item (i.e. consumer-facing price promotions, not retailer-facing promotions). Multiple-unit pricing offers (e.g. buy-one-get-one-free, 3 for the price of 2 etc.) shall be included in line with previous literature [2, 3]. We will only consider price promotions that are available to all consumers, we will not consider discounts or promotions in which eligibility is determined by personal characteristics of the shopper (e.g. student discounts) or previous shopping behaviour (e.g. discounts received due to loyalty-card reward points) or cash-back offers (item sold for normal price and discount received as cash-back).

COMPARATOR(S)/CONTROL

The comparator for research question 1, 'what is the prevalence of price promotions on foods available to purchase in food retail settings', will be the total number of foods (in the paper's defined population) that do not carry price promotions i.e. this may be the total number of foods available to purchase in a supermarket (Powell, 2016), or if an article examines the prevalence of price promotions in a particular food group (e.g. breakfast cereals), then the comparator would be the total number of foods examined in that food group (e.g. total number of breakfast cereals). For studies that examine multiple categories, the results will be entered for each food group.

There are many ways to assess and/or compare the healthiness of foods, for example:

- comparisons between food categories perceived as ‘healthier’ such as fruits and vegetables to food categories perceived as less healthy such as confectionery and soft drinks,
- comparisons within food categories e.g. healthy and unhealthy breakfast cereal (Potvin Kent, Rudnicki and Usher, 2017)
- comparisons based on nutritional composition
- comparisons based on pre-established nutritional composition indicators, such as traffic light labels, foods with and without health symbols, etc.

Therefore, for the second research question, ‘are price promotions more likely to be found on healthy or less healthy foods?’, we will not impose a definition of ‘healthier’ or ‘less healthy’ foods instead we will follow the article’s definitions and compare these definitions in the review.

PRIMARY OUTCOMES

The prevalence of price promotions, overall, and by food group.

SECONDARY OUTCOMES

The difference in prevalence of price promotions between healthier and less healthy (as defined by the article) food categories.

DATA EXTRACTION (SLECTION AND CODING)

References will be imported into Endnote. A single researcher will complete the first title screen to remove any duplicate references. Titles that are clearly unrelated to the primary or secondary research questions will be excluded at this stage.

The abstracts of the remaining references shall then be screened by two researchers, with articles where there are disagreements being taken forward for full review.

The data extraction will be assembled in Excel and the following data will be extracted:

- Study details: authors, year of publication, study design,
- Sample: country, retail setting type, sampling methods (if applicable), data collection methods,
- Data: prevalence of foods carrying price promotions, food/food groups studied,
- Intervention: type of price promotion (proportional discount, multi-purchases i.e. buy-one-get one free, 3 for the price of 2)
- Analyses: statistical analyses performed, whether adjusted for confounding factors
- Results for research questions

RISK OF BIAS (QUALITY) ASSESSMENT

A systematic review of assessment tools used for assessing the quality of observational epidemiology was used to identify an appropriate risk of bias tool. Durant's (1994) Survey Designs and Cross Sectional Studies was adapted for use in this systematic review [10]. The following changes were made as the criterion were not appropriate or relevant to this systematic review:

- Criterion h referring to random sampling (Criterion h),
- Criterion m referring to participant and double blinding, and
- criterion n referring to participant/data exclusions.

Table 1: Criteria used to assess included studies, adapted from Durant's (1994) Survey Designs and Cross Sectional Studies

**Criteria**	**Assessment**
Definition of population	Are the criteria for inclusion of s*hops* described?
	Are the criteria for inclusion of *foods* described?
Sampling strategy	Was the sample drawn randomly from the population or is the sample a complete audit of the population?
Description of sample:	Has the study sample been clearly described:<br>• sample size of foods;<br>• geographic region;<br>• sample size of shops?
Definition of outcome variable	Has the study provided a definition of 'price promotions'?
	Has the method of categorising foods with 'price promotions' been validated (including inter-rater reliability)?
Definition of exposure variables:	Has the study provided a definition of any included exposure variables (e.g. food categories, healthiness of foods)?
	Has the method of categorising foods for exposure variables been validated (including inter-rater reliability)?

There were three possible outcomes for each paper: Meets criterion; does not meet criterion; not enough info to make assessment.

STRATEGY FOR DATA SYNTHESIS

For research question 1, a two-step strategy will be used. First a sign test that indicates if the study answers the primary research question 'do price promotions increase purchasing intentions'? The

second step will be to quantify these effects. From the initial scoping review we expect there to be much heterogeneity in the study design (for example, different types of price promotion and/or different food categories). Therefore a random effects meta-analysis will be performed to generate a weighted average effect size from the included studies.

For research question 2, we will conduct a sign-test that indicates if the prevalence of price promotions on healthy foods is higher or lower than for unhealthy foods, and whether or not the difference is statistically significant (measured with the threshold of $p<0.05$). As we expect there to be much heterogeneity in the way studies differentiate between healthy and less healthy foods we will not conduct a meta-analyses.

ANALYSIS OF SUBGROUPS OR SUBSETS

For research question 1 we will perform analyses by food group. If data are sufficient we shall conduct analyses by type of promotion.

For research question 2 we will perform analyses for healthier vs less healthy food groups.

DISSEMINATION PLANS

We will present the findings from this review in an article to be submitted to a peer-reviewed publication.

REFERENCES

1. Nakamura R, Suhrcke M, Jebb SA *et al.* (2015) Price promotions on healthier compared with less healthy foods: a hierarchical regression analysis of the impact on sales and social patterning of responses to promotions in Great Britain. *American Journal of Clinical Nutrition* 101, 808-816.
2. Powell LM, Kumanyika SK, Isgor Z *et al.* (2016) Price promotions for food and beverage products in a nationwide sample of food stores. *Preventive Medicine* 86, 106-113.
3. Taillie LS, Ng SW, Xue Y *et al.* (2017) Deal or no deal? The prevalence and nutritional quality of price promotions among US food and beverage purchases. *Appetite* 117, 365-372.
4. Ayala GX, D'Angelo H, Gittelsohn J *et al.* (2017) Who is behind the stocking of energy-dense foods and beverages in small stores? the importance of food and beverage distributors. *Public health nutrition* 20, 3333-3342.
5. Caspi CE, Lenk K, Pelletier JE *et al.* (2017) Association between store food environment and customer purchases in small grocery stores, gas-marts, pharmacies and dollar stores. *International Journal of Behavioral Nutrition and Physical Activity* 14.
6. Ravensbergen EAH, Waterlander WE, Kroeze W *et al.* (2015) Healthy or Unhealthy on Sale? A cross-sectional study on the proportion of healthy and unhealthy foods promoted through flyer advertising by supermarkets in the Netherlands. *Bmc Public Health* 15.
7. Thornton LE, Crawford DA, Lamb KE *et al.* (2017) Where do people purchase food? A novel approach to investigating food purchasing locations. *International Journal of Health Geographics* 16.

8. The World Bank (2018) World Bank Country and Lending Groups. https://datahelpdesk.worldbank.org/knowledgebase/articles/906519-world-bank-country-and-lending-groups
9. Reardon T & Hopkins R (2006) The supermarket revolution in developing countries: Policies to address emerging tensions among supermarkets, suppliers and traditional retailers. *The European journal of development research* 18, 522-545.
10. DuRant RH (1994) Checklist for the evaluation of research articles. *The Journal of adolescent health : official publication of the Society for Adolescent Medicine* 15, 4-8.
