# Supplementary material for: A systematic review, and meta-analysis, examining the prevalence of price promotions on foods and whether they are more likely to be found on less-healthy foods
Source: Public Health Nutr. 2020 Mar 25;23(8):1281–96. doi: 10.1017/S1368980019004129 (PMC7196736; doi:10.1017/S1368980019004129)
Supplement: Supplementary file 1 [file S1368980019004129sup.zip › S1368980019004129sup002.pdf]

Appendix B: Search terms

A SYSTEMATIC REVIEW, AND META-ANALYSIS, EXAMINING THE PREVALENCE OF PRICE PROMOTIONS ON FOODS AND WHETHER THEY ARE MORE OR LESS LIKELY TO BE FOUND ON UNHEALTHY FOODS.

Database	Query	Items found	Date of search	Updated Query	Items found	Date of search
PubMed	Search (((((((pric* promotion* OR promotional OR discount OR buy one get one free OR free OR two for one OR three for two OR 2 for 1 OR 3 for 2 OR three for one OR 3 for 1 OR advertisements OR flyers OR leaflets OR special offer))) AND ((food OR food supply OR grocery OR groceries OR meat OR poultry OR beef OR lamb OR pork OR fruit OR veg OR vegetables OR cereal OR oats OR oatmeal OR porridge OR cereals OR bread OR wheat OR rye OR barley OR cheese OR milk OR butter OR dairy OR beverage OR beverages OR fish OR shellfish))) AND ((Supermarket* OR Store* OR Grocery OR Mart OR shop OR convenience))) AND ((cross-sectional OR cross sectional OR crosssectional OR observation OR observational Or retrospective OR audit OR dataset OR data))) NOT (((drug OR pharmeceutical* OR pheromone* OR quargel OR mobile applications OR apps OR embryo OR feces OR faeces OR campylobacter OR campylobacteriosis OR staphylococcus OR staphylococcal OR salmonella OR antimicrobials OR suicide OR criminal OR crime* OR victimisation OR periodontal OR birth OR nerves OR nervous OR hepatitis OR outbreak OR pituitary OR soil OR snail OR diarrhea OR inflammation OR anemia OR anaemia)))) AND ("2000"[Date - Create] : "2018"[Date - Create])	2415	11/05/ 2018	Search (((("2018/05/11"[Date - Entrez] : "3000"[Date - Entrez])) OR ("2018/05/11"[Date - Create] : "3000"[Date - Create]))) AND (((price promotion* OR promotional OR discount* OR "buy one get one free" OR free OR "two for one" OR "three for two" OR "2 for 1" OR "3 for 2" OR "three for one" OR "3 for 1" OR advertisements OR flyers OR leaflets OR special offer*) AND (food OR "food supply" OR grocery OR groceries OR meat OR poultry OR beef OR lamb OR pork OR fruit OR veg OR vegetables OR cereal OR oats OR oatmeal OR porridge OR cereals OR bread OR wheat OR rye OR barley OR cheese OR milk OR butter OR dairy OR beverage OR beverages OR fish OR shellfish) AND (Supermarket* OR Store* OR Grocery OR Mart OR shop OR convenience) AND (cross-sectional OR cross sectional OR crosssectional OR observation OR observational Or retrospective OR audit OR dataset OR data)) NOT (drug OR pharmeceutical* OR pheromone* OR quargel OR "mobile applications" OR apps OR embryo OR feces OR faeces OR campylobacter OR campylobacteriosis OR staphylococcus OR staphylococcal OR salmonella OR antimicrobials OR suicide OR criminal OR crime* OR victimisation OR periodontal OR birth OR nerves OR nervous OR hepatitis OR outbreak OR pituitary OR soil OR snail OR diarrhea OR inflammation OR anemia OR anaemia))	189	20/06/ 2019

Database	Query	Items found	Date of search	Updated Query	Items found	Date of search
Web of Science	Search (((((((pric* promotion* OR promotional OR discount OR buy one get one free OR free OR two for one OR three for two OR 2 for 1 OR 3 for 2 OR three for one OR 3 for 1 OR advertisements OR flyers OR leaflets OR special offer))) AND ((food OR food supply OR grocery OR groceries OR meat OR poultry OR beef OR lamb OR pork OR fruit OR veg OR vegetables OR cereal OR oats OR oatmeal OR porridge OR cereals OR bread OR wheat OR rye OR barley OR cheese OR milk OR butter OR dairy OR beverage OR beverages OR fish OR shellfish))) AND ((Supermarket* OR Store* OR Grocery OR Mart OR shop OR convenience))) AND ((cross-sectional OR cross sectional OR crosssectional OR observation OR observational Or retrospective OR audit OR dataset OR data))) NOT (((drug OR pharmeceutical* OR pheromone* OR quargel OR mobile applications OR apps OR embryo OR feces OR faeces OR campylobacter OR campylobacteriosis OR staphylococcus OR staphylococcal OR salmonella OR antimicrobials OR suicide OR criminal OR crime* OR victimisation OR periodontal OR birth OR nerves OR nervous OR hepatitis OR outbreak OR pituitary OR soil OR snail OR diarrhea OR inflammation OR anemia OR anaemia)))) AND ("2000"[Date - Create] : "2018"[Date - Create])	3075	11/05/ 2018	**#3: #1 NOT #2**<br><br>#1: TS=((price promotion* OR promotional OR discount* OR "buy one get one free" OR free OR "two for one" OR "three for two" OR "2 for 1" OR "3 for 2" OR "three for one" OR "3 for 1" OR advertisements OR flyers OR leaflets OR special offer*) AND (food OR "food supply" OR grocery OR groceries OR meat OR poultry OR beef OR lamb OR pork OR fruit OR veg OR vegetables OR cereal OR oats OR oatmeal OR porridge OR cereals OR bread OR wheat OR rye OR barley OR cheese OR milk OR butter OR dairy OR beverage OR beverages OR fish OR shellfish) AND (Supermarket* OR Store* OR Grocery OR Mart OR shop OR convenience) AND (cross-sectional OR cross sectional OR crosssectional OR observation OR observational Or retrospective OR audit OR dataset OR data))<br><br>#2: ts=(drug OR pharmeceutical* OR pheromone* OR quargel OR "mobile applications" OR apps OR embryo OR feces OR faeces OR campylobacter OR campylobacteriosis OR staphylococcus OR staphylococcal OR salmonella OR antimicrobials OR suicide OR criminal OR crime* OR victimisation OR periodontal OR birth OR nerves OR nervous OR hepatitis OR outbreak OR pituitary OR soil OR snail OR diarrhea OR inflammation OR anemia OR anaemia)	119	20/06/ 2019

Database	Query	Items found	Date of search	Updated Query	Items found	Date of search
Scopus	(TITLE-ABS-KEY (price AND promotion* OR discount*) OR TITLE-ABS-KEY (buy AND one AND get AND one AND free OR promotional OR in-store AND promotions OR advertisements OR flyers OR leaflets) AND TITLE-ABS-KEY (food OR diet OR nutrition OR meat OR dairy OR fish) OR TITLE-ABS-KEY (poultry OR fruit OR fruits OR vegetables OR veg OR vegetable) OR TITLE-ABS-KEY (beef OR lamb OR pork OR bread OR wheat OR rye OR barley) OR TITLE-ABS-KEY (cheese OR milk OR butter OR beverage OR beverages OR fish OR shellfish)	1233	14/05/ 2018	(TITLE-ABS-KEY (price AND promotion* OR promotional OR discount* OR "buy one get one free" OR free OR "two for one" OR "three for two" OR "2 for 1" OR "3 for 2" OR "three for one" OR "3 for 1" OR advertisements OR flyers OR leaflets OR "special offer*") AND TITLE-ABS-KEY (food OR "food supply" OR grocery OR groceries OR meat OR poultry OR beef OR lamb OR pork OR fruit OR veg OR vegetables OR cereal OR oats OR oatmeal OR porridge OR cereals OR bread OR wheat OR rye OR barley OR cheese OR milk OR butter OR dairy OR beverage) AND TITLE-ABS-KEY (supermarket* OR store* OR grocery OR mart OR shop OR convenience) AND TITLE-ABS-KEY (cross-sectional OR cross AND sectional OR crosssectional OR observation OR observational OR retrospective OR audit OR dataset OR data)) AND (LIMIT-TO (PUBYEAR , 2019) OR LIMIT-TO (PUBYEAR , 2018))	11	20/06/ 2019
