# Supplementary material for: A systematic review, and meta-analysis, examining the prevalence of price promotions on foods and whether they are more likely to be found on less-healthy foods
Source: Public Health Nutr. 2020 Mar 25;23(8):1281–96. doi: 10.1017/S1368980019004129 (PMC7196736; doi:10.1017/S1368980019004129)
Supplement: Supplementary file 1 [file S1368980019004129sup.zip › S1368980019004129sup003.pdf]

Appendix C: Data extraction sheet column headings

A SYSTEMATIC REVIEW, AND META-ANALYSIS, EXAMINING THE PREVALENCE OF PRICE PROMOTIONS ON FOODS AND WHETHER THEY ARE MORE OR LESS LIKELY TO BE FOUND ON UNHEALTHY FOODS.

PAPER DETAILS		Paper ID
		Author (Year)
		Full reference
ELIGIBILITY		Did the study measure price promotion prevalence in a retail setting?
		Did the study measure difference (in price promotion prevalence) between healthy and unhealthy foods, in a retail setting?
		Reason for exclusion
STUDY DETAILS		Country
		Region, Country
		WHO income category
		Study design
		Data (collection/type)
STUDY DEFINITIONS		of price promotions
		of healthy and unhealthy foods
DETAILS ABOUT SAMPLE OF FOODS		Sample - shops
		What type of shops?
		Random sample?
		Sample-foods
		What type of foods?
		Comments - sample
DATA COLLECTION DETAILS		Data collection methods
		Data collection time period
		Type of promotional data
		Type of promotional data - Comments
Systematic review analyses	RESEARCH QUESTION 1 ANALYSES	Analysis: RQ1
		FOODS - Proportion on Promotion
	RESEARCH QUESTION 2 ANALYSES	Measure of healthfulness
		What was the difference?
		Was difference significant? ($p<0.05$)
		Analysis
		Comments
		Notes
Risk of bias	Definition of population	criteria for inclusion of shops described
		criteria for inclusion of foods described?
	Sampling strategy	Was the sample drawn randomly from the population or is the sample a complete audit of the population?
	Description of sample:	Description of size of foods? y/n
		Description of geographic region? y/n
		Description of sample size of shops
	Definition of outcome variable (price promotion):	Definition of price promotions? y/n
		Has the method of categorising foods with ‘price promotions’ been validated (including inter-rater reliability)? y/n
	Definition of potential exposure variables:	Definition of exposure variables? y/n
		Has the method of categorising foods for exposure variables been validated (including inter-rater reliability)? y/n
